# Sustainable Discovery of Natural Anti-Aging Bioactives from Food Resources: Current Status and Machine Learning Perspectives

**DOI:** 10.3390/cimb48070703

**Published:** 2026-07-10

**Authors:** Zhangziyan Zhao, Shanxue Jiang, Haishu Sun

**Affiliations:** School of Light Industry Science and Engineering, Beijing Technology and Business University, Beijing 100048, China; 2305010232@st.btbu.edu.cn (Z.Z.); jiangshanxue@btbu.edu.cn (S.J.)

**Keywords:** natural products, food-derived bioactives, anti-aging, computational discovery, machine learning

## Abstract

Existing anti-aging drugs are often limited by toxicity and resistance. In contrast, natural substances derived from food resources, edible plants, and agricultural by-products offer advantages such as low toxicity and suitability for dietary intake. Utilizing these resources aligns with sustainable development goals by promoting the valorization of food waste and functional food development; however, their complex composition makes traditional discovery inefficient and resource-intensive. Machine learning (ML) provides a powerful, sustainable in silico solution. By analyzing vast datasets, computational models can rapidly screen thousands of candidates, significantly reducing the chemical waste and time associated with traditional wet-lab screening. This review focuses on the current status of food-derived anti-aging bioactives and the emerging ML-based perspectives in this field. Key natural compounds and plant extracts are discussed, highlighting their dietary origins and mechanisms. Furthermore, we explore how advanced algorithms accelerate the identification of novel bioactives. Importantly, we address current translational gaps, including the need for explainable AI, ADME (Absorption, Distribution, Metabolism, and Excretion) prediction, and the standardization of complex mixtures. Overcoming these bottlenecks is essential for the sustainable development of effective, food-based anti-aging ingredients.

## 1. Introduction

Aging arises from multiple interrelated factors, including chronic inflammation, mitochondrial impairment, genomic and epigenetic modifications, and aberrant intercellular signaling [1]. To intervene in these processes, various synthetic anti-aging or senolytic agents have been developed and investigated [2]. Although some of these agents show promising activity in eliminating senescent cells, their broader application may be limited by potential toxicity, off-target effects, and mechanism-dependent resistance. These limitations are particularly relevant when anti-aging strategies rely on single or narrowly defined molecular targets [3]. Therefore, safer and multi-target anti-aging strategies are urgently needed.

Many natural compounds, particularly those derived from food and edible plants, have complex chemical structures and diverse mechanisms of action. For example, procyanidin C1 (found in grape seeds and other fruits) can not only target and clear senescent cells but also induce apoptosis by regulating mitochondrial function and ROS levels without damaging normal cells [4]. Importantly, many of these food-derived compounds, such as quercetin (abundant in apples and onions) and curcumin (the active component of turmeric), are not only bioactive but also possess a history of safe dietary consumption. This makes them prime candidates for development into functional food ingredients or nutraceuticals aimed at supporting healthy aging [5].

Food-derived botanical extracts are important sources of anti-aging agents; however, their highly diverse chemical structures and complex matrices make traditional screening inefficient, costly, and low-throughput. Such complexity creates a major bottleneck in identifying active components from edible plants and agricultural by-products [6,7]. In this context, machine learning (ML) provides a green and sustainable computational strategy. By training models on food-derived metabolite libraries and known bioactivity datasets, ML can rapidly prioritize potential active candidates from large chemical spaces, thereby narrowing the experimental scope. This in silico pre-screening approach reduces the consumption of reagents, minimizes chemical waste, and accelerates the sustainable discovery of natural anti-aging agents [8,9].

Given the increasing application of ML in discovering bioactive compounds, including those with anti-aging potential, a timely review of this field is needed. This review discusses representative food-derived natural compounds with anti-aging activities, followed by an overview of edible plant extracts exhibiting anti-aging properties. We then focus on the potential of ML algorithms in accelerating the discovery of such dietary anti-aging bioactives. This review aims to provide insights for future research on the discovery of novel bioactive ingredients from food sources for functional food and nutraceutical applications.

## 2. Literature Search Strategy

This manuscript is a narrative review rather than a systematic review or scoping review. The aim was to summarize representative food-derived natural compounds, edible plant/fungal extracts, and machine learning approaches relevant to anti-aging bioactive discovery. The literature search was primarily conducted in Web of Science. The search terms included “natural products”, “food-derived bioactives”, “anti-aging”, “cellular senescence”, “senolytics”, “antioxidant”, “plant extracts”, “functional foods”, “machine learning”, “graph neural network”, “random forest”, and “XGBoost”. Publications from approximately 2015 to 2025 were considered, with emphasis on recent studies from the past ten years. Studies were included if they reported anti-aging, anti-senescence, antioxidant, anti-inflammatory, or machine-learning-based screening evidence related to food-derived compounds or edible natural extracts. Studies were excluded if they were unrelated to food-derived resources, lacked experimental or computational relevance, or were not available in English.

## 3. Aging, Current Challenges, and Advantages of Food-Derived Bioactives

Aging is a complex biological process characterized by progressive functional decline at the molecular, cellular, tissue, and organismal levels. It is closely associated with cellular senescence, mitochondrial dysfunction, oxidative stress, chronic low-grade inflammation, impaired proteostasis, telomere attrition, altered nutrient-sensing pathways, and extracellular matrix degradation. These processes contribute to the development of age-related diseases, including cardiovascular diseases, neurodegenerative disorders, metabolic diseases, osteoporosis, skin aging, and cancer. Therefore, anti-aging intervention aims not only to extend lifespan but also to improve healthspan and delay the onset of age-associated disorders [10].

Current anti-aging strategies include synthetic senolytics, senomorphics, caloric restriction mimetics, antioxidants, anti-inflammatory agents, and lifestyle interventions. However, despite promising preclinical data, the clinical translation of many synthetic agents remains hampered by underwhelming trial outcomes, a lack of standardized evaluation methodologies, and insufficient evidence of sustained efficacy in humans. Moreover, aging is regulated by highly interconnected pathways across multiple biological scales, and current interventions often target a single hallmark within a narrow scope, which is insufficient to fully address the complex aging phenotype [11].

Food-derived bioactive compounds are promising candidates for healthy-aging interventions. Compared with non-edible medicinal materials or purely synthetic compound libraries, food sources usually have a long history of dietary exposure and are more suitable for long-term intake [12]. This is particularly important because anti-aging interventions are often preventive and chronic. Foods contain diverse bioactive molecules, including polyphenols, flavonoids, anthocyanins, carotenoids, terpenoids, amino acid derivatives, peptides, and polysaccharides. These compounds can regulate multiple aging-related pathways, such as AMPK/mTOR, PI3K/Akt, Nrf2/HO-1, NF-κB, SIRT-mediated pathways, autophagy, apoptosis, and mitochondrial function [13].

In addition, food-derived bioactives are compatible with the development of functional foods, nutraceuticals, dietary supplements, functional beverages, fortified foods, and cosmeceutical ingredients. The use of agricultural and food-processing by-products, such as fruit peels, seeds, leaves, bran, and plant residues, can also improve resource utilization and reduce waste, supporting sustainable development and a circular economy [14]. Therefore, food resources provide a practical, relatively safe, consumer-acceptable, and sustainable platform for discovering anti-aging ingredients. However, their chemical complexity, variable composition, limited stability, and low bioavailability remain major challenges. To address these issues, systematic extraction, chemical characterization, bioactivity evaluation, and ML-assisted prediction are needed to accelerate the discovery and development of food-derived anti-aging bioactives [15].

## 4. Selected Food-Derived Compounds with Anti-Aging Activities

Nature offers a vast repository of structurally diverse molecules with profound biological activities. In recent years, the paradigm of anti-aging research has increasingly shifted towards dietary interventions and functional foods due to their excellent safety profiles and long-term suitability. Food-derived bioactive compounds, particularly those extracted from edible plants, marine resources, and agricultural by-products, have demonstrated remarkable potential in modulating aging-related pathways (e.g., AMPK, mTOR, and SIRT1) and mitigating cellular senescence. Table 1 lists various natural substances with anti-cellular-senescence properties. This section provides a brief discussion of these compounds.

### 4.1. Anthocyanin

Anthocyanins are a common class of natural compounds abundant in pigmented fruits and vegetables such as berries, grapes, red cabbage, and purple sweet potatoes, with anti-inflammatory and antioxidant properties. Anthocyanins belong to the polyphenol class of compounds and can react with metal ions to block the catalytic activity of active metal ions, inhibit free radical production, and exert antioxidant, antimutagenic, and anticancer effects. It has been reported that anthocyanins have a wide range of biological activities. A study demonstrated the ability of anthocyanins to reduce the proliferation of cancer cells depending on the molecular type, concentration, and cell line used [16]. In an experiment using galactose-induced senescence as the model, anthocyanins were found to inhibit the PI3K/Akt/mTOR signaling pathway and induce apoptosis in the senescent cells [17].

### 4.2. Fisetin

Fisetin is a flavonoid abundant in many kinds of fruits and vegetables, such as apples, strawberries, and onions, and has many benefits, such as antioxidant, anti-inflammatory, and anti-tumor effects [18]. It is formed by a 15-carbon skeleton and two benzene rings connected by a pyran ring. Compared with other structures, its extraordinary antioxidant activity depends on the number and configuration of its hydroxyl groups [19].

Studies have shown that PPARγ is related to cellular senescence, that the mTORC2 signaling pathway regulates autophagy and cellular senescence, and that fisetin exerts anti-aging effects by activating PPARγ and inhibiting mTORC2 in VSMCs [20]. According to previous reports, fisetin reduces cellular ROS levels, increases resistance to oxidative stress, and can significantly inhibit the degeneration of dopamine neurons. Dietary supplements containing 8 to 500 mg of fisetin are commercially available and advertised for anti-aging and anti-inflammatory effects [21]. Zhao et al. (2023) investigated the anti-aging effects of fisetin by inhibiting the expression of Stc1, thereby suppressing the Akt signaling pathway and inducing apoptosis in senescent cells [22].

Fisetin has consistently been reported to have limited water solubility and low bioavailability in reviews, preclinical studies, and human pharmacokinetic research. Its low systemic exposure is due not only to poor solubility but also to rapid metabolism, enzymatic degradation, and P-glycoprotein-mediated efflux after oral intake. As a result, the parent compound is quickly converted into sulfate and glucuronide conjugates [23].

### 4.3. Curcumin

Curcumin is a powerful antioxidant. It is a polyphenol that can neutralize free radicals, remove harmful oxidants, protect cells from oxidative damage, improve the body’s antioxidant capacity, help slow aging, and prevent chronic diseases. Curcumin can interact simultaneously with many molecular targets, including receptors, growth factors, kinases, transcription factors, enzymes, adhesion molecules, apoptotic regulators, pro-inflammatory factors, and other proteins. Through these interactions, curcumin can influence multiple biological processes, such as redox homeostasis, inflammation, proliferation, migration, apoptosis, and wound healing, thereby potentially improving memory and delaying aging and age-related diseases [24].

In Bielak-Zmijewska’s study, researchers adjusted the curcumin concentration to be similar to the serum curcumin concentration after dietary intake, and their laboratory results showed that curcumin could exert anti-aging effects by altering the levels of proteins involved in the cellular aging process [25]. It has been reported that loss of *ahr-1* promotes the health and longevity of nematodes under basal conditions. This empirical research was conducted in nematodes and showed that curcumin significantly and reproducibly extended the lifespan and healthspan of nematodes in an *ahr-1*-dependent manner [26].

Curcumin shows potential anti-aging activity, but its application is limited by poor bioavailability. Due to its low water solubility, limited intestinal absorption, chemical instability, and rapid intestinal/hepatic metabolism, the plasma and tissue levels of free curcumin remain very low after oral intake [27]. Most curcumin is rapidly converted into glucuronide, sulfate, and reduced metabolites and eliminated in urine or feces. Therefore, although curcumin is generally considered safe at dietary levels, its high-dose or long-term use requires further safety evaluation, and improved delivery systems are needed to enhance its anti-aging applicability [28].

### 4.4. Ergothioneine

Ergothioneine (EGT), a sulfur-containing histidine derivative mainly found in mushrooms, black beans, oat bran, and certain meats, is a dietary antioxidant with potential anti-aging activity [29]. In a high-glucose-induced endothelial senescence model, endothelial cells were pretreated with EGT at 0.01–1.00 mM for 12 h and then exposed to 25 mM glucose for 48 h. EGT showed no obvious cytotoxicity, and 0.5 mM EGT exhibited the strongest protective effect. It reduced ROS production and decreased the proportion of SA-β-gal-positive senescent cells from approximately 44.2% to 28.3%. Mechanistically, EGT restored SIRT1 and SIRT6 expression and downregulated p66Shc and NF-κB. Inhibition of SIRT1 or silencing of SIRT6 abolished this protective effect, suggesting that EGT alleviates endothelial senescence partly through the SIRT1/SIRT6 pathway [30].

EGT was also studied in a UVB-induced photoaging model using a keratinocyte/fibroblast co-culture system. Keratinocytes were pretreated with EGT at 0.1, 1, or 10 mM for 2 h and then exposed to 20 mJ/cm^2^ UVB. EGT reduced ROS accumulation and apoptosis, suppressed caspase-8 and PARP cleavage, and decreased the levels of inflammatory cytokines such as TNF-α, IL-1β, and IL-6. It also prevented the downregulation of Nrf2, HO-1, and HSP70. In co-cultured fibroblasts, EGT reduced SA-β-gal staining and MMP-1/CCN1 expression while increasing type I collagen expression, indicating an indirect protective effect against fibroblast senescence and collagen degradation [31].

Ergothioneine can also alleviate UVB damage in keratinocytes by activating the Nrf2/HO-1 pathway, thereby reducing induced fibroblast senescence [32]. These effects are consistent with the antioxidant, metal-chelating, and cytoprotective properties previously reported for EGT [33]. However, these mechanistic findings are mainly derived from in vitro cellular models. Although some animal studies have reported protective effects of EGT in oxidative stress-related conditions, direct in vivo validation of the SIRT1/SIRT6 or Nrf2/HO-1 pathways in anti-aging or anti-senescence models remains limited. Therefore, these pathway-related conclusions should be interpreted primarily as cell-based mechanistic evidence, and further animal studies are needed to confirm whether these mechanisms operate in vivo [34].

### 4.5. Quercetin

Quercetin is a flavonol abundant in common foods like onions, capers, apples, berries, and tea, with well-reported antioxidant and anti-aging properties [35]. Its strong free-radical-scavenging activity is mainly attributed to structural features such as the catechol group in the B ring and the hydroxyl group at position 3. These structural features contribute to its antioxidant capacity and may help protect red blood cell membrane proteins, including Na^+^/K^+^-ATPase, thereby slowing age-related changes in red blood cells [36]. Quercetin can activate AMP-activated protein kinase (AMPK), thereby inducing apoptosis and attenuating oxidative stress-induced senescence in vascular smooth muscle cells [37]. Quercetin is a proteasomal activator with antioxidant properties that can increase the lifespan of HFL-1 primary human fibroblasts [38]. Quercetin has been studied as a therapeutic agent for neurodegeneration, diabetes, cancer, and inflammation. Studies have indicated that quercetin plays a vital role in the prevention of age-related diseases, and quercetin’s anti-aging properties may be leveraged in the future. Its widespread dietary presence makes it a promising candidate for development as a functional food ingredient or supplement for age-related health support [39].

Quercetin has limited oral bioavailability and undergoes extensive phase II metabolism after intake. Although it is generally considered safe at standard supplemental doses, available safety data for long-term high-dose use remain limited. In addition, high concentrations of quercetin may exert pro-oxidant or cytotoxic effects in certain cellular contexts, and potential concerns have been raised regarding gastrointestinal discomfort, nervous system-related effects, and interactions with concomitant medications [40,41].

### 4.6. Tannic Acid

**Figure 1:**
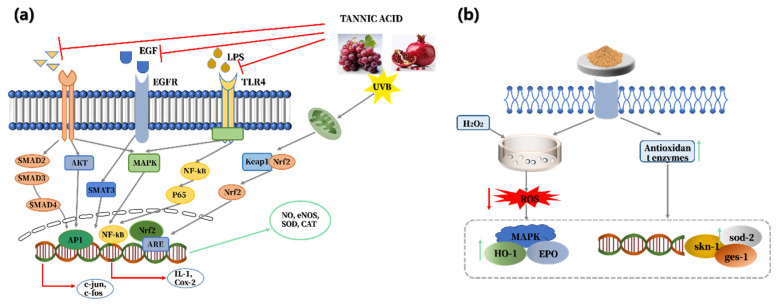
Anti-aging mechanisms of (**a**) tannic acid and (**b**) *Lycium barbarum* leaf extracts. Adapted from refs [43,81].

Tannic acid is a naturally occurring polyphenolic compound that is widely found in various trees and higher plant species, as well as in foods and beverages, such as green tea, coffee, and fresh fruits [42]. Studies have shown that tannic acid can help fibroblasts resist photoaging by reducing UVB-induced oxidative stress and inhibiting the expression of elastase and collagenase (Figure 1) [43]. In another study, the potential radical scavenging activity of tannins was evaluated using three radical scavenging methods, namely 2,2′-azino-bis(3-ethylbenzothiazoline-6-sulfonic acid) (ABTS), 1,1-diphenyl-2-picryl-hydrazyl (DPPH), and superoxide anion scavenging assays. The results showed that tannic acid has a strong ability to scavenge free radicals, which may help delay cellular aging [44].

### 4.7. Chlorogenic Acid

Chlorogenic acid is a polyphenolic compound present in *Coffea arabica* beans. It exhibits anti-aging properties by reducing the expression of key matrix metalloproteinases (MMPs) in the skin and inhibiting the production of reactive oxygen species (ROS) in dermal cells [45]. In an experiment, mouse fibroblast cell line was used as a model to simulate the ultraviolet (UV)-induced skin aging environment. In vitro intervention was performed with chlorogenic acid, and the activities of MMPs and xanthine oxidase, as well as the synthesis of type-I procollagen, were assessed. The results showed that chlorogenic acid can significantly downregulate the protein and gene expression of MMPs, reduce collagen and elastin degradation in the skin extracellular matrix, and prevent skin elasticity loss and wrinkles formation. Additionally, chlorogenic acid can inhibit the activity of xanthine oxidase, thereby reducing the accumulation of ROS and alleviating oxidative stress-induced damage to skin cells. Furthermore, chlorogenic acid effectively promoted the synthesis of type-I procollagen, which helps maintain the stability of skin structure and enhances skin firmness. Through these pathways, chlorogenic acid plays a role in delaying the senescence of skin cells in vitro [15]. Another experimental study indicated that chlorogenic acid maintains skin firmness and preserves the integrity of the skin matrix by regulating collagen metabolism. By scavenging ROS, repairing DNA damage, and inhibiting apoptosis, chlorogenic acid also protected against UVA-induced skin photoaging in vitro and slowed skin cell senescence [46].

### 4.8. β-Carotene

β-Carotene is a carotenoid widely found in fruits, vegetables, flowers, algae, and some seafood. Direct evidence for its anti-senescent activity was reported in a study using both mesenchymal stem cell (MSC)-based in vitro models and aged mice [7].

Zheng et al. (2022) provided direct evidence that β-carotene exerts anti-senescent effects in MSC-based models and aged mice [47]. In vitro, β-carotene attenuated H_2_O_2_-induced senescence in adipose- and bone marrow-derived MSCs and replicative senescence in serially passaged adipose-derived MSCs. These effects were associated with reduced SA-β-gal positivity, downregulation of p16, p21, and p53, improved proliferation and S-phase entry, and decreased DNA damage, inflammatory signaling, and oxidative stress. The study further suggested that KAT7–P15 signaling may be involved in β-carotene-mediated protection against MSC senescence. In 22-month-old C57 mice, oral β-carotene improved cognitive, anxiety-related, and motor behaviors and reduced tissue inflammation, senescence-associated staining, injury, and fibrosis. However, the study did not directly verify whether the KAT7–P15 mechanism observed in MSCs also operated in vivo [47].

In addition, β-carotene-induced improvement of mitochondrial structure/function and reduction in senescence-associated secretory phenotype (SASP) release were reported in independent experimental systems. Therefore, these effects should be considered model-specific and complementary rather than directly comparable outcomes from a single experimental model [48,49].

### 4.9. D-Limonene

D-Limonene is a monoterpene with a C10 carbon skeleton, predominantly derived from citrus fruits and Pinaceae plants [50]. D-limonene exerts potent anti-aging and skin-rejuvenating effects, primarily by alleviating oxidative stress and inhibiting chronic inflammation. It significantly reduces the levels of pro-inflammatory cytokines and the oxidative stress marker malondialdehyde (MDA) while enhancing the activities of antioxidant enzymes. Meanwhile, D-limonene promotes the synthesis of type I and type III collagen, increases skin thickness, and restores normal skin histological structure. These effects are achieved by regulating oxidative and inflammatory biomarkers, supporting its potential as both a preventive and therapeutic anti-aging agent [51]. In addition to the aforementioned anti-aging mechanisms, D-limonene can mitigate UV-induced skin photoaging by inhibiting the activity of MMPs [52]. In another study, D-limonene, a natural monoterpenoid abundant in the essential oil of *Zanthoxylum acanthopodium* DC, was reported to exert anti-aging effects on skin, mainly by inhibiting key enzymes associated with skin aging. Through molecular docking, D-limonene was shown to stably bind to collagenase, elastase, hyaluronidase, and tyrosinase, thereby effectively suppressing the activities of these enzymes. Consequently, it reduces the degradation of collagen and elastin, maintains hyaluronic acid content, and alleviates excessive melanin deposition, ultimately improving aging-related skin symptoms such as wrinkles, sagging, dryness, and hyperpigmentation. Furthermore, D-limonene complies with Lipinski’s Rule of Five and exhibits favorable human intestinal absorption, good bioavailability, and high safety without mutagenic or carcinogenic risks, making it a safe and effective natural active ingredient for skin anti-aging [53].

Although oxidative stress is an important contributor to cellular damage and age-related functional decline, antioxidant activity alone should not be considered direct evidence of anti-aging efficacy. Many studies evaluate antioxidant capacity using chemical assays such as DPPH, ABTS, or ferric reducing antioxidant power (FRAP), which do not fully reflect cellular redox regulation, bioavailability, metabolism, or long-term physiological effects in vivo [54]. Therefore, antioxidant activity should be interpreted as a potential mechanistic contributor rather than definitive proof of delayed aging. Stronger evidence requires demonstration of effects on validated aging-related endpoints, such as senescence markers, SASP factors, DNA damage, mitochondrial function, tissue function, lifespan, healthspan, or clinical aging biomarkers [55].

Overall, the anti-aging mechanisms of these food-derived compounds can be summarized into several major categories. First, many compounds, such as anthocyanins, quercetin, chlorogenic acid, tannic acid, and ergothioneine, exert antioxidant effects by reducing ROS accumulation or activating antioxidant signaling pathways. Second, several compounds regulate inflammation and SASP-related responses, thereby reducing chronic low-grade inflammation. Third, compounds such as fisetin, quercetin, and curcumin modulate key aging-related pathways, including AMPK, mTOR, PI3K/Akt, SIRT, and autophagy-related signaling. Finally, some compounds, particularly chlorogenic acid, tannic acid, D-limonene, and carotenoids, protect extracellular matrix integrity by reducing collagen degradation, inhibiting MMPs, or supporting mitochondrial function. Therefore, these compounds act through multi-target and pathway-level regulation rather than a single mechanism.

**Table 1:** Pure natural compounds with anti-aging properties.

Compound	Classification	Anti-Aging Mechanism	Dose	Evidence Level	Ref.
Anthocyanin	Flavonoid	Inhibits PI3K/Akt/mTOR signaling, reduces senescence markers, and enhances autophagy, mitochondrial function, and antioxidant capacity.	In vitro: 40–160 μg/mL, 72 h; in vivo: 50–200 mg/kg/day, 8 weeks.	In vitro and animal.	[17]
Fisetin	Flavone	Reduces senescence markers, fibrosis, Akt signaling, and Bcl-2; promotes senescent cell apoptosis.	Fisetin 500 mg/kg in feed + 20 mg/kg gavage, 3 times/week, 7 weeks.	Animal.	[22]
Curcumin	Polyphenols	Exhibits hormetic anti-aging effects by regulating antioxidant, stress-response, inflammatory, mTOR/AMPK, sirtuin, autophagy, and gut microbiota pathways.	*Caenorhabditis elegans*: 20–200 μM; *Drosophila*: 50–500 μM.	Animal.	[25]
Quercetin	Flavonol	Enhances proteasome activity and protein homeostasis; reduces oxidative damage and senescence markers.	Quercetin 2–5 μg/mL; quercetin–caprylate 0.5–10 μg/mL.	In vitro.	[38]
Ergothioneine	Amino acid	Activates Nrf2/HO-1 and SIRT1/SIRT6; scavenges ROS; suppresses apoptosis and inflammation	0.1–10 mM, 2 h pretreatment + 48 h treatment 0.01–1 mM; 12 h pretreatment + 48 h treatment	In vitro.	[30,31]
Tannic acid	Polyphenols	Antioxidant; inhibits collagenase and elastase; suppresses MMP-1 expression; reduces ROS, lipid peroxidation, DNA damage, and mitochondrial depolarization.	1–5 µM, 1 h pretreatment + UVB (600 mJ/cm^2^), 24 h incubation.	In vitro.	[56]
Chlorogenic acid	Polyphenols	Targets ENO1; inhibits glycolysis; reduces senescence markers (p16/p21), SASP (TNF-α, IL-6, IL-1β), and ROS; alleviates skin photoaging.	In vitro: 10 µM, UVA 10 J/cm^2^, 24 h; in vivo: 25 and 100 mg/kg/day, oral gavage, 8 weeks.	In vitro and animal.	[57]
β-Carotene	Terpenoids	Regulates KAT7–P15 signaling and reduces senescence, inflammation, oxidative stress, and DNA damage.	In vitro: 0.5–5 µM; in vivo: 0.5 mg/mouse/day, oral gavage.	In vitro and animal.	[47]
D-limonene	Terpenoids	Reduces inflammatory cytokines (TNF-α, TGF-β1), angiogenesis, and oxidative stress; increases antioxidant enzymes.	25 and 50 mg/kg; rat model.	Animal.	[58]

## 5. Anti-Aging Properties of Selected Edible Plant and Fungal Extracts

### 5.1. Cortex mori

*Cortex mori* is derived from the root bark of mulberry (*Morus alba*), a plant whose leaves are used to feed silkworms and whose fruits (mulberries) are consumed as food. *Cortex mori* contains a variety of polyphenols, flavonoids and polysaccharides with strong antioxidant and anti-aging properties [59]. In a mouse model of chronic obstructive pulmonary disease, injection of *Cortex mori* extract significantly reduced the levels of aging-related marker proteins and decreased the expression of p53 mRNA, suggesting that *Cortex mori* extract may inhibit fibroblast senescence through the PI3K/Akt pathway [60]. In another study on skin anti-aging, *Cortex mori* extract was found to regulate the AGE-RAGE/MAPK signaling pathway to slow down skin cell aging by inhibiting mitogen-activated protein kinases. In an experiment on the effects of *Cortex mori* extract on cell replication, it was found that *Cortex mori* extract promoted cell replication by reducing the acetylation levels of p53 protein and histone proteins, limiting DNA damage and increasing the nucleus size to achieve anti-aging effects [5].

### 5.2. Rhodiola rosea

*Rhodiola rosea* has a history of use as an adaptogen in traditional medicine and is commonly consumed as a dietary supplement or herbal tea. The chemical components of *Rhodiola rosea* extract include salidroside, polysaccharide, and flavonoids, which have antioxidant, anti-aging, anti-cancer and neuroprotective effects [61]. In *Drosophila melanogaster*, 30 mg/mL *Rhodiola rosea* extract significantly extended the mean lifespan from 25.0 to 28.5 days in males and from 26.9 to 30.1 days in females; the mean lifespan differences were 3.50 ± 0.53 and 3.21 ± 0.68 days, respectively, based on 95% confidence intervals [62]. It was also found that the effect of *Rhodiola rosea* extract on lifespan was independent of the signaling pathways associated with caloric restriction but dependent on the ratio of protein to carbohydrates in the diet and the expression of Msn2/Msn4 and Yap1 regulatory proteins [63]. By adding *Rhodiola rosea* extract to keratinocyte culture medium for a period of time and performing quantitative analysis, scientists found that the keratinocytes treated with *Rhodiola rosea* extract had increased telomerase activity and fewer senescent cells [64]. However, most studies have only confirmed that *Rhodiola rosea* extract can prolong the lifespan of adult fruit flies and it does not significantly affect any secondary physiological mechanisms that may lead to artificial lifespan extension [62]. While there are many different kinds of *Rhodiola rosea* extract, further research is needed to standardize extracts and fully evaluate their safety profile for human consumption.

### 5.3. Cortex moutan

*Cortex moutan* belongs to the Peony family, which comprises ornamental, edible, and medicinal plants originating in China. *Cortex moutan*, an important Chinese medicine, has anti-inflammatory, antibacterial and anti-tumor effects. Phytochemical studies have shown that phenols and their glycosides, monoterpenes, glycosides, flavonoids, polysaccharides, organic acids, amino acids, and volatile oils have been isolated and identified in *Cortex moutan* extract [65]. Scientists studying the activity of *Cortex moutan* extract against H_2_O_2_-induced senescence in an MRC-5 cell model found that *Cortex moutan* extract attenuated cellular senescence by reducing SA-β-gal activity and the expression of other aging biomarkers [29]. In an in-depth study of the effects of *Cortex moutan* extract on human bronchial epithelial cells, it was found that treatment with *Cortex moutan* extract reduced the levels of ROS inside and outside the cell and inhibited mitogen-activated kinase signaling, slowing down cell aging [66]. In another study, researchers found that *Cortex moutan* extract may be able to inhibit cell senescence by activating the Nrf2 transcription factor signaling pathway to enhance cellular antioxidant activity [67]. Its dual status as a medicinal and edible plant makes it an interesting candidate for exploring dietary interventions for aging.

### 5.4. Pine Needle

Pine needles have a history of use in folk medicine and are sometimes used to make herbal teas or infusions. The main ingredients in pine needle essential oil consist of higher aldehydes, terpenes, and terpene esters [68]. Researchers isolated 20 compounds from Pinus morrisonicola needles using bioactivity-guided fractionation. Several flavonoids and lignans, including kaempferol derivatives and matairesinol, exhibited antioxidant activity, while compounds 3, 4, and 5 significantly inhibited MMP-2 in HT-1080 cells, suggesting potential anti-aging effects [69]. In a study on the anti-aging activity of an extract of pine needles, the experimenter extracted the essential oil of pine needles by hydrodistillation and tested its anti-aging activity. The results showed that the essential oil in the pine needle extract had significant radical-scavenging and reducing properties that were concentration-dependent, with the essential oil removing 21.17% of ABTS free radicals at 0.16 μg/mL and nearly 100% at 2.5 μg/mL [70]. In future studies, the purification of pine needle extract can be further investigated; additional experiments should be carried out so that pine needle extract can be used as an anti-aging ingredient for disease treatment [71].

### 5.5. Acmella oleracea

*Acmella oleracea*, also known as the toothache plant or paracress, is used both medicinally and as a pungent leafy vegetable in some cuisines (e.g., Brazilian dishes like jambú). *Acmella oleracea* is a plant with great potential, belonging to the Asteraceae family, and the main ingredient in its extract, spilanthol, is the main metabolite responsible for its anesthetic and muscle-relaxing effects. Using physical methods, scientists tested the composition of *Acmella oleracea* extract, as well as its antioxidant capacity. By using a method including a 2,2-diphenyl-1-trinitrophenylhydrazine free radical scavenging test, *Acmella oleracea* extract was determined to be high in flavonoids, which have strong antioxidant properties and are used in cosmetics to fight ageing and reduce wrinkles [72]. In Franz diffusion cell tests using human skin, *Acmella oleracea* extract was shown to be able to cross the epidermal barrier to reach the dermis and beyond, inhibiting subcutaneous muscle contraction and slowing down cellular aging [29]. Scientists found that spilanthol in *Acmella oleracea* extract exhibited neurorelaxant activity in zebrafish embryos, suggesting that it has potential to be used as an anti-wrinkle component at the subcutaneous tissue level with anti-aging potential [73]. Its edible nature also prompts investigation into its potential as a functional food ingredient.

### 5.6. Cordyceps

*Cordyceps* is an edible mushroom with potential medicinal benefits. Its extract contains a variety of bioactive compounds, including exopolysaccharides and cordycepin, which have been used as anti-tumor, immunomodulatory, antioxidant, and gonadotropic agents [74]. In the course of studying *Cordyceps* extract’s antioxidant and anti-aging properties, it was found that when the *Cordyceps* extract concentration was 1 mg/mL, its low-molecular-weight CMP and polysaccharides had significant DPPH, ABTS, and hydroxyl radical scavenging activities, which were 13.71%, 38.33% and 23.94%, 30.36%, 69.32%, and 43.82%, respectively. From these data, it can be seen that *Cordyceps* extract has antioxidant and anti-aging effects [75]. A study found that the cordycepin in the extract inhibited cell senescence by inducing cell apoptosis and inhibiting the mTOR signaling pathway [76]. *Cordyceps* extract has been evaluated using the DPPH assay and ROS clearance bioactivity of the cell. The experimental results of a study on the properties of the antioxidant activity of a hot-water *Cordyceps* extract showed a greater, non-statistically significant antioxidant effect compared to a mycelium extract [77]. Based on a study of the effect of *Cordyceps* extract on metabolic disease, it has the potential to improve mitochondrial function and enhance energy metabolism while increasing oxygen utilization in cells, contributing to its anti-aging effects [78]. Existing studies have shown that use of *Cordyceps* extract results in significant increases in sirtuin expression, NAD^+^ synthesis, ATP production, ROS clearance, and collagen synthesis. Future research directions could focus on advancing the understanding of the mechanism of action of *Cordyceps* extract, identifying other molecular targets involved, optimizing its efficacy, and extending the duration of relatively short clinical trials to overcome the limitations of existing studies [79].

In this review, cellular senescence-related endpoints, including senescent-cell apoptosis, ROS reduction in cellular models, and modulation of aging-associated pathways such as mTOR, sirtuins, and NAD^+^ metabolism, are considered more direct evidence of anti-aging activity. Chemical antioxidant assays such as DPPH and ABTS are treated as supportive evidence of antioxidant potential rather than primary anti-aging endpoints.

### 5.7. Lycium barbarum Leaves

*Lycium barbarum* (commonly known as wolfberry) is an important medicinal plant in China, the fruits, leaves, and root bark of which are rich in bioactive compounds believed to be beneficial to human health. Its fruits, known as goji berries, are a popular superfood and dietary supplement worldwide, and its leaves are also consumed as a vegetable or tea in some regions. Studies of the effects of *Lycium barbarum* leaf extract on photoaged human dermal fibroblasts and their mechanisms of action have found that *Lycium barbarum* leaf extract can inhibit oxidative stress and apoptosis, promote cell proliferation and protein levels associated with the skin’s extracellular matrix, and participates in anti-photoaging in a concentration-dependent manner [80]. In experiments exploring its anti-aging effects, scientists first purified *Lycium barbarum* leaf extract using polyamide resins, characterized it via ultra-high performance liquid chromatography–mass spectrometry, and subsequently applied it to hydrogen peroxide (H_2_O_2_)-treated human umbilical vein endothelial cells (HUVECs) and *Caenorhabditis elegans* (*C. elegans*) to observe and compare changes in the cells. The results showed that the *Lycium barbarum* leaf extract attenuated H_2_O_2_-induced HUVEC apoptosis, decreased ROS and malondialdehyde production levels, and increased superoxide dismutase, glutathione peroxidase, and catalase activities. At the same time, *Lycium barbarum* leaf extract upregulated the expression of *sod-2*, *gcs-1*, and *skn-1* genes, prolonging the life span of *C. elegans*. These results suggest that *Lycium barbarum* leaf extract acts as an antioxidant for anti-aging purposes [81]. In another study investigating its anti-aging activity, researchers treated senescent endothelial progenitor cells with 10, 25, and 50 mg/L of *Lycium barbarum* leaf extract. The results showed that the extract enhanced telomerase activity and may inhibit endothelial progenitor cell senescence through activation of the PI3K/Akt pathway [82].

### 5.8. Green Tea

The main components of green tea extract are polyphenols, catechins and amino acids, which have strong antioxidant and anti-inflammatory properties [83]. To study the effects of green tea extract on aging cardiomyocytes, researchers set up aerobic exercise training experiments, using green tea extract and the combination of two groups of controlled experiments on aging rat cardiomyocytes as the research object, to observe and compare changes in the apoptosis markers of aging rat cardiomyocytes. Results indicated that the mice exposed to green tea extract exhibited decreased levels of aging markers at 12 weeks. Mice fed green tea extract exhibited reduced free radicals and increased antioxidant enzyme activity in liver tissue at 7 days, which delayed aging. In another study, when green tea extract was fed to mice undergoing a UV-mediated photoaging process, significant increases in hydroxyproline levels were observed in vitro. Catalase activity increased with a decrease in protein carbonyl content, and the results suggested that green tea extract could increase levels of collagen and elastin fibers and reduce expression of the collagen-degrading MMP-3 enzyme, thus showing potential anti-wrinkle effects [84]. In experiments investigating the effects of green tea extract on the brain, the substance EGCG, the main catechin in green tea, was found to activate nerve cells and reduce age-related decline in cognitive function. Theanine and arginine in green tea extract have been found to inhibit stress-related life span reduction [85]. These studies collectively affirm green tea extract as a dietary-source anti-aging agent with broad potential for incorporation into functional foods and beverages.

### 5.9. Ginkgo biloba

While the leaf extract of *Ginkgo biloba* is primarily used medicinally, the seeds (ginkgo nuts) are a traditional food item in East Asian cuisine. The proven biological effects of *Ginkgo biloba* extract are scavenging free radicals, reducing oxidative stress, and reducing nerve damage. The extract contains flavonoids and terpenoids such as quercetin, kaempferol, isorhamnetin, ginkgolide, and dihydrofolate, which have powerful antioxidant properties [86].

In a study on the anti-aging activity of *Ginkgo biloba* extract, it was found to inhibit ROS and MMP-1 degradation in hemodialysis filtration (HDF) due to its high levels of flavonoids and lactones. Ginkgolide A and ginkgolide have better collagen-promoting activities and should be further investigated for their anti-aging activities [87]. The effects of EGb761 in *Ginkgo biloba* extract were studied in an experiment using senescence heart cells as a model. The results showed that EGB761 reduced aging-related proteins and improved the diastolic function of heart cells. It was speculated that EGb761 might improve the main calcium pump for the sarcoplasmic reticulum to recover calcium ion function by increasing the amount of PLN phosphorylation at Ser16, thereby improving the diastolic capacity of heart cells and slowing down the aging process [88]. Experiments have shown that *Ginkgo biloba* extract can delay cell senescence and prevent age-related diseases. However, existing clinical trials are heterogeneous, as different dosage forms, dosages, and dosing times have been studied. For these reasons, it is necessary for future experiments to investigate *Ginkgo biloba* extract to determine the dosage, drug form, and duration of treatment necessary to achieve a preventive effect or act as a therapeutic adjuvant under aging conditions [89].

### 5.10. Red algae

Of all seaweeds, *Red algae*, considered the original species in the phylogenetic tree, is a macroalgae that adheres to rocks in the intertidal and light-coastal zones and has been used for years as a dietary supplement for healthy living. Red algae extracts contain various bioactive components, including sulfated polysaccharides, phycobiliproteins, mycosporine-like amino acids, polyphenols, and peptides [90]. Fucoidan, however, is more commonly associated with brown algae rather than red algae; therefore, the discussion of fucoidan has been revised and separated from the *Red algae* section [91].

In a study of Fucoidan’s anti-aging properties, it was found that *Fucoidan* reduced the aging of endothelial colony-forming cells in long-term culture by reducing the activity of age-related beta-galactosidase [92]. In addition, other studies have found that polysaccharides from algae can decrease the expression of pro-aging protein p21 but increase the expression of anti-aging protein regucalcin, which has a significant anti-aging effect [93]. In order to study the effect of *Red algae* extract as a cosmetic raw material on skin photoprotection, researchers irradiated the skin of mice treated with *Red algae* extract with UV light. The study found that the *Red algae* extract contained mycosporine-like amino acids that could absorb UVA radiation. In addition, the antioxidant substances contained in the extract may reduce the damage caused by ROS. It was concluded that *Red algae* extract had anti-aging properties [94]. These properties support its use not only in cosmetics but also as a source of marine-derived bioactive compounds for nutraceuticals.

The anti-aging mechanisms, major bioactive compounds, application prospects, and supporting references of the selected edible plant and fungal extracts discussed in this section are summarized in Table 2.

The extraction method strongly influences the yield, composition, purity, and biological activity of food-derived anti-aging compounds. Conventional extraction methods include solvent extraction, maceration, reflux extraction, and Soxhlet extraction. These methods are simple and widely used but often require long extraction times and high solvent consumption and may cause degradation of thermolabile compounds [97].

In recent years, greener and more efficient extraction technologies have attracted increasing attention, including ultrasound-assisted extraction, microwave-assisted extraction, enzyme-assisted extraction, supercritical fluid extraction, pressurized liquid extraction, and deep eutectic solvent-based extraction. These methods can improve extraction efficiency, reduce solvent use, and better preserve bioactive compounds such as polyphenols, flavonoids, carotenoids, polysaccharides, and peptides [98].

## 6. Machine Learning Approaches for Anti-Aging Ingredient Discovery

The compounds and extracts summarized above demonstrate that food resources provide structurally diverse candidates with multiple anti-aging mechanisms. However, this diversity also creates major challenges for conventional discovery, including complex chemical compositions, variable extraction efficiency, unclear active components, and high experimental cost. These limitations provide a strong rationale for introducing ML into the discovery pipeline. ML can connect food sources, extraction parameters, chemical structures, bioactivity data, and aging-related endpoints, thereby improving the prioritization of candidate compounds and optimizing experimental design. Although the direct application of ML specifically to anti-aging food bioactives is still an emerging field (Table 3), ML has already been extensively and successfully applied in discovering related functional properties, such as antioxidant and anti-inflammatory activities, which are core mechanisms of anti-aging.

### 6.1. Representative ML Approaches and Applications

In one study, 2352 compounds were screened in an etoposide-induced senescence model, and 45 active senolytic compounds were identified after senescence was confirmed by SA-β-gal staining and p16, p21, and KI67 mRNA expression. A graph neural network (GNN) model was then trained to predict senolytic activity. The model achieved an area under the precision–recall curve (auPRC) of 0.24, which was markedly higher than the random baseline of 0.019 because active compounds accounted for only approximately 1.9% of the dataset. After screening more than 800,000 compounds, followed by structural filtering and experimental validation, 25 of 216 high-ranking candidates were confirmed as active, corresponding to a working hit rate of 11.6%. Three representative compounds showed selective senolytic activity, partly through Bcl-2 inhibition. Initial toxicity assays suggested favorable safety profiles, and one compound, BRD-K56819078, reduced senescent-cell burden in aged mouse kidneys. Further studies are needed to evaluate their pharmacokinetics, long-term safety, and clinical applicability [99].

Researchers used ML to screen peptides, extracted unstructured text data related to anti-aging and the extracellular matrix from the literature through natural language processing (NLP), and combined it with structured peptide data to construct a training dataset. They used graph analysis techniques to transform peptide features into graph structures, trained supervised learning models to predict anti-aging activity, and, through three rounds of iterations of prediction-test-optimization, selected peptide pep_35E7UW. The researchers verified through in vitro and ex vivo experiments that the peptide exerts its anti-aging effect by regulating the ECM synthesis pathway and cell migration ability. Further studies, including oral bioavailability assessments, are needed to evaluate its potential for dietary interventions [106].

**Figure 2:**
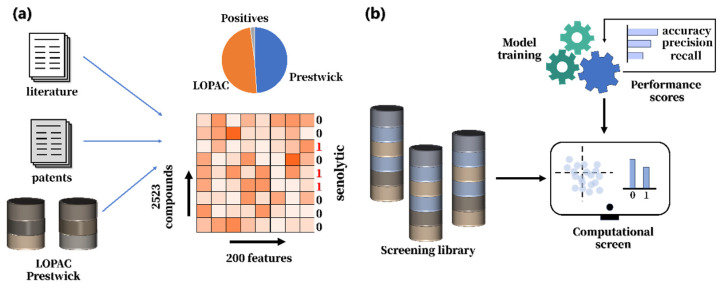
Machine learning approach of (**a**) training pipeline and (**b**) performance metrics. Adapted from ref [9].

Researchers used the XGBoost ensemble learning model to study 58 anti-aging compounds (positive samples), integrating and predicting 2465 non-anti-aging compounds (negative samples) and screening out 21 high-probability compounds. Three effective anti-aging drugs, namely Ginkgetin, Oleandrin, and Periplocin, were identified through validation of anti-aging effects using both oncogene-induced aging (OIS) and treatment-induced aging (TIS) models. The specific process is illustrated in Figure 2. However, the model used in that study relied on a limited number of positive samples; not only are more heterogeneous data needed to improve generalization, but in vivo toxicity also needs to be further verified [9].

Researchers utilized models such as graph neural networks (GNNs) and random forests to analyze molecular structural features, predicting anti-aging activity from tens of thousands of compounds. Single-cell RNA sequencing combined with deep learning was employed to identify senescence-specific targets, while data from model organisms like *Drosophila* was used to train models for predicting the age of cells [99,107].

### 6.2. Current Challenges and Bottlenecks

While ML holds transformative potential for discovering anti-aging ingredients from food sources, its systematic application in this domain faces several interconnected bottlenecks that hinder translation from prediction to practical application.

First, data scarcity and model limitations pose a fundamental constraint. The development of robust ML models relies on large, high-quality datasets. However, publicly available, aging-specific bioactivity data for food-derived compounds remain limited. This scarcity leads to small-scale training sets, which can result in models with sample bias and poor generalization when applied to the vast structural diversity of natural products. Furthermore, the black-box nature of many advanced models (e.g., deep neural networks) limits mechanistic interpretation. While such models can predict activity, they often cannot elucidate the underlying molecular interactions or dynamic binding mechanisms, making it difficult to gain actionable biological insights or assess specific toxicity risks based on prediction alone. To address this black-box problem, interpretable AI methods should be incorporated into future ML workflows. SHAP and LIME can quantify the contribution of molecular descriptors or structural fragments to model predictions, while attention-based visualization in graph neural networks can highlight key atoms, bonds, or functional groups associated with predicted anti-aging activity. These approaches can improve model transparency and help link computational predictions with plausible biological mechanisms.

Second, the inherent complexity of food and botanical extracts introduces unique challenges for both discovery and standardization. Unlike single synthetic drugs, natural extracts (e.g., *Cortex mori*, *Rhodiola rosea*) are complex mixtures of polyphenols, flavonoids, and other components. This complexity creates a dilemma between purity and bioactivity/toxicity. On one hand, impurities from incomplete extraction may introduce unintended effects, such as enhancing mitochondrial toxicity (as seen with some curcumin extracts) or triggering oxidative stress. On the other hand, excessive purification to isolate a single compound may alter its activity or ignore crucial synergistic effects present in the whole extract, and high concentrations of pure substances (e.g., quercetin) may themselves exhibit cytotoxic effects through pathways like excessive autophagy induction.

Third, a significant gap exists in translational validation, creating a major attrition point. Currently, there is a stark disconnect between computational prediction and subsequent experimental validation relevant to food or supplement development. It is estimated that only a few ML-predicted anti-aging compounds will progress to animal studies, with the vast majority remaining at the in vitro stage. This gap leaves critical questions relating to functional food application, such as oral bioavailability, tissue distribution, long-term safety under dietary administration, and efficacy in physiologically relevant aging models, unanswered. For instance, compounds like ginkgo biflavones may show promise in cellular senescence models, but data on their pharmacokinetics or organ-specific toxicity in aged organisms are lacking.

### 6.3. Future Perspectives and Strategies

To bridge the gaps identified above and realize the full potential of ML in discovering food-derived anti-aging ingredients, a concerted effort across the following strategic directions is essential.

First, the foremost task is to address the data bottleneck by building high-quality, aging-specific databases. Rather than a general call to action, specific steps must be taken to integrate existing food-specific databases (such as FooDB and Phenol-Explorer) with established pharmacological databases (like ChEMBL and PubChem). Researchers should focus on curating datasets that explicitly link food compound structures with validated senescence markers (e.g., SA-β-gal activity, SASP secretion). To overcome data scarcity, cross-domain data integration is necessary. Food-specific databases such as FooDB and Phenol-Explorer should be combined with broader chemical and pharmacological resources, including PubChem, ChEMBL, DrugBank, and BindingDB. Such integration can link dietary sources, molecular structures, physicochemical properties, target annotations, ADME/toxicity data, and senescence-related endpoints, thereby expanding training datasets and improving model generalization.

Second, future efforts are needed to overcome the challenges posed by complex food matrices requires a shift from viewing them as problems to leveraging them as information-rich sources. Multi-omics integration combining transcriptomics, metabolomics, and proteomics can map the global biological responses to food extracts, identifying key senescence-modulating pathways and potential synergistic effects between components. This systems-level understanding should be coupled with computational simulations, such as molecular dynamics, to model interactions between food bioactives and their molecular targets at an atomic level. These in silico insights can guide more focused and intelligent wet-lab experiments, reducing reliance on blind screening.

Finally, a paradigm shift in validation is needed to bridge the gap between computational hits and applicable ingredients. This involves developing a stepwise, food-relevant validation cascade. After in vitro confirmation of activity, critical steps include assessing bioaccessibility and bioavailability using simulated gastrointestinal models and evaluating efficacy and safety through dietary intervention studies in physiologically relevant animal models of aging (e.g., naturally aged mice). The ultimate step requires human intervention trials that measure not only safety but also changes in validated biomarkers of aging and healthspan following consumption of the candidate ingredient in a feasible dietary format. This pipeline ensures that promising ML predictions are rigorously tested for real-world application in functional foods or nutraceuticals.

## 7. Conclusions

In summary, the rich diversity of food-derived anti-aging compounds and extracts presents a valuable and relatively safe resource for promoting healthy aging. However, their discovery and development are hindered by the intrinsic complexity of natural matrices and the inefficiency of conventional screening. ML is emerging as a transformative tool to address these bottlenecks. By enabling the rapid and intelligent prioritization of candidates from vast chemical libraries, ML has demonstrably increased screening hit rates by an order of magnitude. This acceleration is crucial for efficiently translating the potential of edible plants and foods into viable candidates for functional ingredients.

Despite this promise, several significant limitations must be acknowledged. Clinical evidence remains scarce, as the vast majority of existing findings derive from in vitro or animal models rather than rigorous human intervention trials with validated aging biomarkers. Bioavailability also poses a major barrier, as many promising compounds suffer from poor solubility and rapid metabolism, raising questions about whether effective tissue concentrations are achievable through dietary intake. Toxicological evaluation remains insufficient, particularly for long-term, high-dose supplementation in aging populations with polypharmacy and altered metabolic capacities. Furthermore, translating results from simplified cellular senescence models to the complex, multifactorial nature of human aging is inherently challenging, and most studies overlook the potential synergistic or antagonistic interactions among compounds in whole-food matrices. To fully realize the potential of ML-driven discovery, future efforts must prioritize the construction of high-quality, aging-specific bioactive datasets, the integration of ADME and toxicity prediction into screening pipelines, and the establishment of robust, food-centric validation cascades, from bioaccessibility assessment to dietary intervention studies in aged animal models and, ultimately, human trials. Success in this interdisciplinary endeavor will depend on sustained collaboration among computational scientists, food chemists, and nutritionists, ultimately bridging the gap between computational prediction and the development of effective, safe dietary interventions for healthy aging.

## Figures and Tables

**Table 2:** Natural extracts with anti-aging properties.

Extract	Compounds	Anti-Aging Mechanism	Application Prospects	Reference
*Cortex mori*	Flavonoids	Reduces senescence via PI3K/Akt signaling.	Natural antioxidant with neuroprotective and endocrine-regulatory potential.	[59,60]
*Rhodiola rosea*	Flavonoids	Exerts anti-aging effects by promoting DNA repair and reducing inflammation.	Potential dietary supplement with anti-fatigue and antidepressant effects.	[62,63]
*Acmella oleracea*	Spilanthol/N-alkylamides	Reduces wrinkles by relaxing subcutaneous muscles and may support collagen-related extracellular matrix repair.	Local anesthesia.	[29,72]
Pine needle	Terpenoids, Flavonoids	Exerts antioxidant, anti-inflammatory, and immunomodulatory effects, thereby reducing cellular senescence.	Cosmetics for wrinkle reduction and dietary supplements for healthy aging.	[71]
*Cortex moutan*	Phenols	Enhances antioxidant defenses by increasing SOD and GSH-Px activity and reducing MDA levels.	Cell-protective, anti-inflammatory, whitening, and anti-aging applications.	[65,66]
*Cordyceps*	Fungal metabolites	Inhibits NF-κB signaling, activates caspase-dependent apoptosis, and suppresses DNA synthesis in senescent cells.	Anti-inflammatory and anti-aging applications in skincare formulations.	[76]
*Lycium barbarum* leaves	Flavonoids	Reduce H_2_O_2_-induced oxidative damage by lowering ROS levels, thereby delaying cell senescence.	Antioxidant skincare ingredient and potential functional food component.	[81]
Green tea	Amino acid	Theanine and arginine reduce stress responses and support neuronal health, potentially delaying brain aging.	Dietary supplements or functional foods for stress reduction and healthy brain aging.	[84,85]
*Ginkgo biloba* leaves	Flavone	Reduces ROS accumulation and MMP-1 expression in HDFs, exerting anti-aging effects.	Anti-aging skin care products; health care products to prevent memory decline in the elderly.	[87]
*Red algae*	Flavone	Scavenges DPPH and ABTS radicals and shows strong antioxidant capacity.	Antioxidant cosmetics and potential neuroprotective applications.	[95,96]

**Table 3:** Applications of machine learning in the discovery of anti-aging bioactive compounds.

Machine Learning Method	Dataset Size/Screening Scale	Performance/Key Results	Advantages	Limitations	Ref.
GNN/message-passing graph neural network for predicting senolytic activity from molecular graphs	Initial dataset: 2352 compounds, including 45 actives and 2307 inactives; virtual screening of 804,959 molecules; 266 candidates experimentally tested	auPRC = 0.24 vs. random baseline ≈ 0.019; best Random Forest baseline ≈ 0.15; identified 3 selective senolytics; experimental hit rate ≈ 11.6%	Learns molecular graph structures directly; suitable for large-scale virtual screening; can identify structurally diverse candidates	Very few positives and highly imbalanced data; modest absolute auPRC; many false positives; senolytic activity does not represent all anti-aging activities	[99]
Random Forest feature selection + XGBoost/RF/SVM classification based on RDKit physicochemical descriptors	Training set: 2523 compounds, including 58 senolytics and 2465 negatives; 200 RDKit descriptors reduced to 165 features; screened 4340 compounds	21 candidates tested; discovered 3 senolytics: ginkgetin, oleandrin, and periplocin; screening cost reduced by hundreds-fold	XGBoost works well with small and imbalanced datasets; RF supports feature selection; multiple traditional ML models were compared	Many negatives were assumed inactive, causing possible mislabeling; few and heterogeneous positives; experimental validation remains essential	[9]
XGBoost with fused molecular fingerprints including Morgan, topological, and MACCS fingerprints; PCA/KPCA; Attention-ElixirFP	Extended DrugAge dataset: 1695 small molecules, including 462 positives and 1233 negatives; external databases screened	Attention-ElixirFP 64-bit: Accuracy = 0.849 ± 0.012, ROC AUC = 0.767 ± 0.020; 4 of top 6 candidates extended lifespan in *C. elegans*	Integrates local, topological, and pharmacophore information; XGBoost feature importance helps weight key structural fragments; directly linked to lifespan-extension phenotypes	Depends on known DrugAge compounds and may favor existing structural classes; phenotype-based screening does not directly reveal mechanisms or targets	[100]
Decision Tree, SVM, and KNN using 1D–3D chemical descriptors	Training set: 405 compounds, including 206 reported geroprotectors and 199 compounds without reported geroprotective activity; screened 695,133 natural products from COCONUT	AUC: DT = 0.62, SVM = 0.73, KNN = 0.64; consensus filtering identified 1488 candidate natural-product geroprotectors	Focuses on natural products, highly relevant to anti-aging ingredient discovery; simple and interpretable models; consensus prediction helps reduce false positives; moderate AUC, better suited for candidate ranking; small training set; lacks large-scale wet-lab	Moderate AUC, better suited for candidate ranking; small training set; lacks large-scale wet-lab validation	[101]
SVM, RF, Logistic Regression, MLP, XGBoost, KNN; GAN augmentation; CNN with multi-head attention; Antiaging-FL for anti-aging peptide prediction	After CD-HIT: 220 anti-aging peptides and 220 non-anti-aging peptides; independent test set of 40 peptides; AAP400 used for training; GAN expanded data to 800, and conservative amino-acid substitution up to 4000	Antiaging-FL: AUC = 1.00 on AAP400 and 0.99 on independent test set; ESM-GAN AUC = 0.99/0.95; ESM-CNN AUC = 0.96/0.94; some traditional models reached ACC = 0.975	Covers multiple ML methods with comprehensive metrics; shows ML’s applicability to anti-aging peptide discovery; data augmentation helps address small-sample limitations	Small dataset; uncertain negative definition; potential overfitting and limited generalization; peptide models should not be directly extrapolated to small molecules	[102]
CNN/Deep-SeSMo for phase-contrast image-based senescence recognition and anti-senescent drug screening	Images: 92,242 H_2_O_2_-induced senescent, 41,207 H_2_O_2_ control, 134,097 CPT-induced senescent, and 64,535 CPT control images; screened 80 kinase inhibitors	CNN: Accuracy = 0.93, F1 = 0.88, AUC = 0.98; identified 4 anti-senescent drugs: terreic acid, PD-98059, daidzein, and Y-27632·2HCl	Does not rely on a single molecular target; enables label-free, high-throughput, quantitative phenotype-based screening from cell morphology	Not a molecular-structure-based activity predictor; performance depends on cell type, senescence induction method, and image quality	[103]
Classification Tree-, Random Forest-, and voting-based consensus algorithm using nuclear morphology features	Around 0.1 × 10^6^–0.9 × 10^6^ cells per condition; each training set sampled 10,000 normal and 10,000 treated cells	Evaluated by AUC, ROC, Accuracy, Precision, Recall, and F1; predictions correlated strongly with SA-β-Gal, p21/BrdU, p21/p53 and related markers; useful for identifying senescence-inducing drugs and evaluating senolytics	More lightweight than deep image models; lower computational cost; applicable to cells, tissues, animals, and human samples; supports senotherapy discovery and validation	Mainly detects senescence or supports phenotype-based screening; does not directly predict small-molecule structural activity; lacks a unified QSAR-style metric	[104]
Cascade R-CNN with ResNet/FPN/RPN/GN for bright-field morphology-based single-cell detection and senescence classification	7373 RGB images of 640 × 640 pixels; validation/test sets included tens of thousands of senescent and non-senescent MSC single cells	Replicative senescence detection: mAP = 0.81, AR = 0.93; senescent-cell precision = 0.850, recall = 0.923, F1-score = 0.885; drug-induced senescence: mean precision = 0.896, recall = 0.931, F1-score = 0.924	Automatically detects single cells of different sizes and shapes; supports non-destructive, real-time, scalable MSC senescence detection; useful as an auxiliary tool for anti-aging drug screening	Mainly designed for senescence detection rather than direct compound discovery; morphology transition states may affect classification; primarily validated in MSC senescence	[105]

## Data Availability

No new data were created or analyzed in this study.

## References

[1] Huang X., Li Q., Tao G., Gan X., Lu J., Krasny S., Shi L. (2026). Engineering Extracellular Vesicles for Anti-Aging Therapy: Mechanisms, Applications, and Perspectives. Aging Cell.

[2] Kirkland J.L., Tchkonia T. (2020). Senolytic drugs: From discovery to translation. J. Intern. Med..

[3] Saliev T., Singh P.B. (2025). Targeting Senescence: A Review of Senolytics and Senomorphics in Anti-Aging Interventions. Biomolecules.

[4] Xu Q., Fu Q., Li Z., Liu H., Wang Y., Lin X., He R., Zhang X., Ju Z., Campisi J. (2021). The flavonoid procyanidin c1 has senotherapeutic activity and increases lifespan in mice. Nat. Metab..

[5] Ding A.-J., Zheng S.-Q., Huang X.-B., Xing T.-K., Wu G.-S., Sun H.-Y., Qi S.-H., Luo H.-R. (2017). Current Perspective in the Discovery of Anti-aging Agents from Natural Products. Nat. Prod. Bioprospect..

[6] Abedelmaksoud T.G., Younis M.I., Altemimi A.B., Tlay R.H., Hassan N.A. (2025). Bioactive Compounds of Plant-Based Food: Extraction, Isolation, Identification, Characteristics, and Emerging Applications. Food Sci. Nutr..

[7] Song L., Zhang S. (2023). Anti-Aging Activity and Modes of Action of Compounds from Natural Food Sources. Biomolecules.

[8] Mullowney M.W., Duncan K.R., Elsayed S.S., Garg N., van der Hooft J.J.J., Martin N.I., Meijer D., Terlouw B.R., Biermann F., Blin K. (2023). Artificial intelligence for natural product drug discovery. Nat. Rev. Drug Discov..

[9] Smer-Barreto V., Quintanilla A., Elliott R.J.R., Dawson J.C., Sun J., Campa V.M., Lorente-Macías Á., Unciti-Broceta A., Carragher N.O., Acosta J.C. (2023). Discovery of senolytics using machine learning. Nat. Commun..

[10] Guo J., Huang X., Dou L., Yan M., Shen T., Tang W., Li J. (2022). Aging and aging-related diseases: From molecular mechanisms to interventions and treatments. Signal Transduct. Target. Ther..

[11] Rosen R.S., Yarmush M.L. (2023). Current Trends in Anti-Aging Strategies. Annu. Rev. Biomed. Eng..

[12] Contreras-Pacheco Y.V., Gerardo V.-E., Alberto S.-B.J., Ghotekar S., Fellah M., Larios A.P. (2026). Natural sources of bioactive compounds: Recent advances in isolation, functionalization, and health benefits. Food Res. Int..

[13] Zhu Y.-F., Wu A.-G., Chen M.-Y., Zhou X.-Y., Huang F.-H., Wang L., Yu L., Wen Y.-P., Qin D.-L., Wu J.-M. (2025). Plant-based strategies against aging: Focus on bioactive compounds from medicine-food homology plants. Phytomedicine.

[14] Arshad Z., Shahid S., Hasnain A., Yaseen E., Rahimi M. (2025). Functional Foods Enriched with Bioactive Compounds: Therapeutic Potential and Technological Innovations. Food Sci. Nutr..

[15] Hernandez D.F., Cervantes E.L., Luna-Vital D.A., Mojica L. (2021). Food-derived bioactive compounds with anti-aging potential for nutricosmetic and cosmeceutical products. Crit. Rev. Food Sci. Nutr..

[16] de Arruda Nascimento E., de Lima Coutinho L., da Silva C.J., de Lima V.L.A.G., Aguiar J.d.S. (2022). In vitro anticancer properties of anthocyanins: A systematic review. Biochim. Biophys. Acta (BBA) Rev. Cancer.

[17] Hu X., Yang Y., Tang S., Chen Q., Zhang M., Ma J., Qin J., Yu H. (2023). Anti-Aging Effects of Anthocyanin Extracts of *Sambucus canadensis* Caused by Targeting Mitochondrial-Induced Oxidative Stress. Int. J. Mol. Sci..

[18] Dhar I., Desai K. (2012). Aging: Drugs to Eliminate Methylglyoxal, a Reactive Glucose Metabolite, and Advanced Glycation Endproducts.

[19] Zhang Y., Wu W., Huang C., Lin D. (2025). Fisetin Alleviates d-Galactose-Induced Senescence in C2C12 Myoblasts: Metabolic and Gene Regulatory Mechanisms. J. Proteome Res..

[20] Kim S.G., Sung J.Y., Kang Y.J., Choi H.C. (2023). PPARγ activation by fisetin mitigates vascular smooth muscle cell senescence via the mTORC2-FoxO3a-autophagy signaling pathway. Biochem. Pharmacol..

[21] Park S., Kim B.-K., Park S.-K. (2022). Effects of Fisetin, a Plant-Derived Flavonoid, on Response to Oxidative Stress, Aging, and Age-Related Diseases in *Caenorhabditis elegans*. Pharmaceuticals.

[22] Zhao R., Kou H., Jiang D., Wang F. (2023). Exploring the anti-aging effects of fisetin in telomerase-deficient progeria mouse model. PeerJ.

[23] Krishnakumar I.M., Jaja-Chimedza A., Joseph A., Balakrishnan A., Maliakel B., Swick A. (2022). Enhanced bioavailability and pharmacokinetics of a novel hybrid-hydrogel formulation of fisetin orally administered in healthy individuals: A randomised double-blinded comparative crossover study. J. Nutr. Sci..

[24] Benameur T., Soleti R., Panaro M.A., La Torre M.E., Monda V., Messina G., Porro C. (2021). Curcumin as prospective anti-aging natural compound: Focus on brain. Molecules.

[25] Bielak-Zmijewska A., Grabowska W., Ciolko A., Bojko A., Mosieniak G., Bijoch Ł., Sikora E. (2019). The Role of Curcumin in the Modulation of Ageing. Int. J. Mol. Sci..

[26] Brinkmann V., Romeo M., Larigot L., Hemmers A., Tschage L., Kleinjohann J., Schiavi A., Steinwachs S., Esser C., Menzel R. (2022). Aryl Hydrocarbon Receptor-Dependent and -Independent Pathways Mediate Curcumin Anti-Aging Effects. Antioxidants.

[27] Hegde M., Girisa S., BharathwajChetty B., Vishwa R., Kunnumakkara A.B. (2023). Curcumin Formulations for Better Bioavailability: What We Learned from Clinical Trials Thus Far?. ACS Omega.

[28] El-Saadony M.T.T., Saad A.M., Mohammed D.M., Alkafaas S.S., Ghosh S., Negm S.H., Salem H.M., Fahmy M.A., Mosa W.F.A., Ibrahim E.H. (2025). Curcumin, an active component of turmeric: Biological activities, nutritional aspects, immunological, bioavailability, and human health benefits—A comprehensive review. Front. Immunol..

[29] Liang Y., Su W., Wang F. (2023). Skin Ageing: A Progressive, Multi-Factorial Condition Demanding an Integrated, Multilayer-Targeted Remedy. Clin. Cosmet. Investig. Dermatol..

[30] D’oNofrio N., Servillo L., Giovane A., Casale R., Vitiello M., Marfella R., Paolisso G., Balestrieri M.L. (2016). Ergothioneine oxidation in the protection against high-glucose induced endothelial senescence: Involvement of SIRT1 and SIRT6. Free Radic. Biol. Med..

[31] Ko H.J., Kim J., Ahn M., Kim J.H., Lee G.S., Shin T. (2021). Ergothioneine alleviates senescence of fibroblasts induced by UVB damage of keratinocytes via activation of the Nrf2/HO-1 pathway and HSP70 in keratinocytes. Exp. Cell Res..

[32] Apparoo Y., Phan C.W., Kuppusamy U.R., Sabaratnam V. (2022). Ergothioneine and its prospects as an anti-ageing compound. Exp. Gerontol..

[33] Liu H.-M., Tang W., Wang X.-Y., Jiang J.-J., Zhang W., Wang W. (2023). Safe and Effective Antioxidant: The Biological Mechanism and Potential Pathways of Ergothioneine in the Skin. Molecules.

[34] Paul B.D. (2021). Ergothioneine: A Stress Vitamin with Antiaging, Vascular, and Neuroprotective Roles?. Antioxid. Redox Signal..

[35] Lewińska A., Przybylski P., Adamczyk-Grochala J., Błoniarz D., Litwinienko G., Wnuk M. (2022). Senolysis-Based Elimination of Chemotherapy-Induced Senescent Breast Cancer Cells by Quercetin Derivative with Blocked Hydroxy Groups. Cancers.

[36] Kumar A., Maurya P.K. (2021). Quercetin Mitigates Red Blood Cell Membrane Bound Na+, K+-ATPase Transporter During Human Aging. J. Membr. Biol..

[37] Kim S.G., Sung J.Y., Kim J.-R., Choi H.C. (2020). Quercetin-induced apoptosis ameliorates vascular smooth muscle cell senescence through AMP-activated protein kinase signaling pathway. Korean J. Physiol. Pharmacol..

[38] Chondrogianni N., Kapeta S., Chinou I., Vassilatou K., Papassideri I., Gonos E.S. (2010). Anti-ageing and rejuvenating effects of quercetin. Exp. Gerontol..

[39] Deepika, Maurya P.K. (2022). Health Benefits of Quercetin in Age-Related Diseases. Molecules.

[40] Mirza M.A., Mahmood S., Hilles A.R., Ali A., Khan M.Z., Zaidi S.A.A., Iqbal Z., Ge Y. (2023). Quercetin as a Therapeutic Product: Evaluation of Its Pharmacological Action and Clinical Applications—A Review. Pharmaceuticals.

[41] Silva-Pinto P.A., de Pontes J.T.C., Aguilar-Morón B., Canales C.S.C., Pavan F.R., Roque-Borda C.A. (2025). Phytochemical insights into flavonoids in cancer: Mechanisms, therapeutic potential, and the case of quercetin. Heliyon.

[42] de Oliveira G., Zamataro I.Z., de Oliveira M.S., Gomes A.S., Barros M.G.A., Oliveira A.G., Zanella K., Gonçalves C.C.S. (2024). Development of Cross-Linked Gelatin Hydrogel Films Using Tannic Acid as Anti-Aging Active with Skin Care Potential. J. Braz. Chem. Soc..

[43] Jing W., Xiaolan C., Yu C., Feng Q., Haifeng Y. (2022). Pharmacological effects and mechanisms of tannic acid. Biomed. Pharmacother..

[44] Gülçin I., Huyut Z., Elmastaş M., Aboul-Enein H.Y. (2010). Radical scavenging and antioxidant activity of tannic acid. Arab. J. Chem..

[45] Cho Y.-H., Bahuguna A., Kim H.-H., Kim D.-i., Kim H.-J., Yu J.-M., Jung H.-G., Jang J.-Y., Kwak J.-H., Park G.-H. (2017). Potential effect of compounds isolated from *Coffea arabica* against UV-B induced skin damage by protecting fibroblast cells. J. Photochem. Photobiol. B Biol..

[46] Xue N., Liu Y., Jin J., Ji M., Chen X. (2022). Chlorogenic Acid Prevents UVA-Induced Skin Photoaging through Regulating Collagen Metabolism and Apoptosis in Human Dermal Fibroblasts. Int. J. Mol. Sci..

[47] Zheng W.V., Xu W., Li Y., Qin J., Zhou T., Li D., Xu Y., Cheng X., Xiong Y., Chen Z. (2022). Anti-aging effect of β-carotene through regulating the KAT7-P15 signaling axis, inflammation and oxidative stress process. Cell. Mol. Biol. Lett..

[48] Zhu Z., Sui J., Zhou Y., Hu B., Gong D., Sun G., Xia H. (2025). The Preclinical and Clinical Evidence of Carotenoids on Anti-Aging and Its Underlying Mechanisms: A Narrative Review. Food Rev. Int..

[49] Ogrodnik M. (2021). Cellular aging beyond cellular senescence: Markers of senescence prior to cell cycle arrest in vitro and in vivo. Aging Cell.

[50] Yazaki K., Arimura G.-I., Ohnishi T. (2017). ‘Hidden’ terpenoids in plants: Their biosynthesis, localization and ecological roles. Plant Cell Physiol..

[51] AlBairmani R.J.H., Shihab E.M., Ridha-Salman H., Kadhim H.M., Mohammed R.M., Shahooth S.S., Abdalah M.E., Hameed Z.E., Hajwal S.K., Albasri O.W. (2025). D-limonene attenuates D-galactose-induced skin aging mouse model. J. Mol. Histol..

[52] Proshkina E., Plyusnin S., Babak T., Lashmanova E., Maganova F., Koval L., Platonova E., Shaposhnikov M., Moskalev A. (2020). Terpenoids as Potential Geroprotectors. Antioxidants.

[53] Kintamani E., Batubara I., Kusmana C., Tiryana T., Mirmanto E., Asoka S.F. (2023). Essential Oil Compounds of Andaliman (*Zanthoxylum acanthopodium* DC.) Fruit Varieties and Their Utilization as Skin Anti-Aging Using Molecular Docking. Life.

[54] Shahidi F., Samarasinghe A. (2025). How to assess antioxidant activity? Advances, limitations, and applications of in vitro, in vivo, and ex vivo approaches. Food Prod. Process. Nutr..

[55] Stojanovic B., Jovanovic I., Stojanovic M.D., Stojanovic B.S., Kovacevic V., Radosavljevic I., Jovanovic D., Kovacevic M.M., Zornic N., Arsic A.A. (2025). Oxidative Stress-Driven Cellular Senescence: Mechanistic Crosstalk and Therapeutic Horizons. Antioxidants.

[56] Daré R.G., Nakamura C.V., Ximenes V.F., Lautenschlager S.O. (2020). Tannic acid, a promising anti-photoaging agent: Evidences of its antioxidant and anti-wrinkle potentials, and its ability to prevent photodamage and MMP-1 expression in L929 fibroblasts exposed to UVB. Free Radic. Biol. Med..

[57] He X., Wang C., Zhang Q., Yang T., Guo Q., Wang Y., Guo J., Wang P., Zhang J., Tang H. (2025). Identifying ENO1 as a protein target of chlorogenic acid to inhibit cellular senescence and prevent skin photoaging in mice. Aging Cell.

[58] Razazi A., Kakanezhadi A., Raisi A., Pedram B., Dezfoulian O., Davoodi F. (2024). D-limonene inhibits peritoneal adhesion formation in rats via anti-inflammatory, anti-angiogenic, and antioxidative effects. Inflammopharmacology.

[59] Li C., Peng Y., Tang W., Li T., Gatasheh M.K., Rasheed R.A., Fu J., He J., Wang W.-D., Shen Y. (2022). Antioxidant, anti-lipidemic, hypoglycemic and antiproliferative effects of phenolics from Cortex Mori Radicis. Arab. J. Chem..

[60] Zeng X.-X., Gao D.-D., Zhang F. (2024). Cortex Mori Radicis [*Morus Alba* L. (Moraceae)] extract alleviates senescence via PI3K/Akt signaling in COPD fibroblasts. Phytomedicine.

[61] Zhuang W., Yue L., Dang X., Chen F., Gong Y., Lin X., Luo Y. (2019). Rosenroot (*Rhodiola*): Potential Applications in Aging-related Diseases. Aging Dis..

[62] Jafari M., Felgner J.S., Bussel I.I., Hutchili T., Khodayari B., Rose M.R., Vince-Cruz C., Mueller L.F. (2007). *Rhodiola*: A Promising Anti-Aging Chinese Herb. Rejuvenation Res..

[63] Li Y., Pham V., Bui M., Song L., Wu C., Walia A., Uchio E., Smith-Liu F., Zi X. (2017). *Rhodiola rosea* L.: An Herb with Anti-Stress, Anti-Aging, and Immunostimulating Properties for Cancer Chemoprevention. Curr. Pharmacol. Rep..

[64] Toh W.H., Chen C.-B., Clapper J., Hsu C.-C., Lee H.-E., Chung W.-H. (2021). Evaluating the Anti-aging Effects of Chinese Herbal Medicine *Rhodiola rosea* L. in Cultured Keratinocytes. bioRxiv.

[65] Yang S., Liu X., He J., Liu M. (2021). Insight into Seasonal Change of Phytochemicals, Antioxidant, and Anti-Aging Activities of Root Bark of *Paeonia suffruticosa* (Cortex Moutan) Combined with Multivariate Statistical Analysis. Molecules.

[66] Ekiert H., Klimek-Szczykutowicz M., Szopa A. (2022). *Paeonia* × *suffruticosa* (Moutan Peony)—A Review of the Chemical Composition, Traditional and Professional Use in Medicine, Position in Cosmetics Industries, and Biotechnological Studies. Plants.

[67] Li H., Xie Y.-H., Yang Q., Wang S.-W., Zhang B.-L., Wang J.-B., Cao W., Bi L.-L., Sun J.-Y., Miao S. (2012). Cardioprotective Effect of Paeonol and Danshensu Combination on Isoproterenol-Induced Myocardial Injury in Rats. PLoS ONE.

[68] Liang Z., Yan J., Zhao S., He L., Zhao X., Cai L., You C., Wang F. (2025). Efficient Extraction, Chemical Characterization, and Bioactivity of Essential Oil from Pine Needles. Phytochem. Anal..

[69] Liu T.-W., Hsiao S.-W., Lin C.-T., Hsiao G., Lee C.-K. (2023). Anti-Aging Constituents from *Pinus morrisonicola* Leaves. Molecules.

[70] Zeng W., Zhang Z., Gao H., Jia L., He Q. (2012). Chemical Composition, Antioxidant, and Antimicrobial Activities of Essential Oil from Pine Needle (*Cedrus deodara*). J. Food Sci..

[71] Li B., Shen Y., He Y., Zhang W. (2013). Chemical constituents and biological activities of *Pinus* species. Chem. Biodivers..

[72] Maxim C., Blaga A.C., Cimpoeșu R., Zinicovscaia I., Peshkova A., Danu M., Barna A.S., Suteu D. (2024). Natural Antioxidants from *Acmella oleracea* Extract as Dermatocosmetic Actives. Sci. Pharm..

[73] Feifei W., Wenrou S., Jinyue S., Qiaochu D., Jingjing L., Jin L., Junxiang L., Xuhui L., Xiao L., Congfen H. (2024). Anti-ageing mechanism of topical bioactive ingredient composition on skin based on network pharmacology. Int. J. Cosmet. Sci..

[74] Chiu C.-P., Hwang T.-L., Chan Y., El-Shazly M., Wu T.-Y., Lo I.-W., Hsu Y.-M., Lai K.-H., Hou M.-F., Yuan S.-S. (2016). Research and development of *Cordyceps* in Taiwan. Food Sci. Hum. Wellness.

[75] Kang J.Y., Lee B., Kim C.H., Choi J.H., Kim M.-S. (2022). Enhancing the prebiotic and antioxidant effects of exopolysaccharides derived from *Cordyceps militaris* by enzyme-digestion. LWT.

[76] Nguyen T.Q., Van Pham T., Andriana Y., Truong M.N. (2025). *Cordyceps militaris*-Derived Bioactive Gels: Therapeutic and Anti-Aging Applications in Dermatology. Gels.

[77] Wang Y.-W., Hong T.-W., Tai Y.-L., Wang Y.-J., Tsai S.-H., Lien P.T.K., Chou T.-H., Lai J.-Y., Chu R., Ding S.-T. (2015). Evaluation of an Epitypified *Ophiocordyceps formosana* (Cordyceps s.l.) for Its Pharmacological Potential. Evid.-Based Complement. Altern. Med..

[78] Bai X., Tan T.-Y., Li Y.-X., Li Y., Chen Y.-F., Ma R., Wang S.-Y., Li Q., Liu Z.-Q. (2020). The protective effect of *Cordyceps sinensis* extract on cerebral ischemic injury via modulating the mitochondrial respiratory chain and inhibiting the mitochondrial apoptotic pathway. Biomed. Pharmacother..

[79] Di Lorenzo R., Falanga D., Ricci L., Colantuono A., Greco G., Angelillo M., Nugnes F., Di Serio T., Costa D., Tito A. (2024). NAD-Driven Sirtuin Activation by *Cordyceps sinensis* Extract: Exploring the Adaptogenic Potential to Promote Skin Longevity. Int. J. Mol. Sci..

[80] Fan L., Luan X., Jia Y., Ma L., Wang Z., Yang Y., Chen Q., Cui X., Luo D. (2024). Protective effect and mechanism of *Lycium barbarum* polysaccharide against UVB-induced skin photoaging. Photochem. Photobiol. Sci..

[81] Niu Y., Liao J., Zhou H., Wang C.-C., Wang L., Fan Y. (2022). Flavonoids from *Lycium barbarum* Leaves Exhibit Anti-Aging Effects through the Redox-Modulation. Molecules.

[82] Liu Y., Weng W., Gao R., Liu Y. (2019). New Insights for Cellular and Molecular Mechanisms of Aging and Aging-Related Diseases: Herbal Medicine as Potential Therapeutic Approach. Oxidative Med. Cell. Longev..

[83] Nhung T.T.N., Chau N.T.B., Hien L.T.M., Linh V.T.H., Le Ha N., Dong D.T.A. (2022). Characteristics of sponge cake preserved by green tea extract powder. J. Food Process. Preserv..

[84] Prasanth M.I., Sivamaruthi B.S., Chaiyasut C., Tencomnao T. (2019). A Review of the Role of Green Tea (*Camellia sinensis*) in Antiphotoaging, Stress Resistance, Neuroprotection, and Autophagy. Nutrients.

[85] Unno K., Nakamura Y. (2021). Green Tea Suppresses Brain Aging. Molecules.

[86] Chan P.-C., Xia Q., Fu P.P. (2007). *Ginkgo biloba* Leave Extract: Biological, Medicinal, and Toxicological Effects. J. Environ. Sci. Health Part C.

[87] Wang X., Gong X., Zhang H., Zhu W., Jiang Z., Shi Y., Li L. (2020). In vitro anti-aging activities of *Ginkgo biloba* leaf extract and its chemical constituents. Food Sci. Technol..

[88] Liu J., Wang J., Chen X., Guo C., Guo Y., Wang H. (2012). *Ginkgo biloba* Extract EGB761 Protects against Aging-Associated Diastolic Dysfunction in Cardiomyocytes of D-Galactose-Induced Aging Rat. Oxidative Med. Cell. Longev..

[89] Barbalho S.M., Direito R., Laurindo L.F., Marton L.T., Guiguer E.L., Goulart R.d.A., Tofano R.J., Carvalho A.C.A., Flato U.A.P., Tofano V.A.C. (2022). *Ginkgo biloba* in the Aging Process: A Narrative Review. Antioxidants.

[90] Carpena M., Garcia-Perez P., Garcia-Oliveira P., Chamorro F., Otero P., Lourenço-Lopes C., Cao H., Simal-Gandara J., Prieto M.A. (2023). Biological properties and potential of compounds extracted from red seaweeds. Phytochem. Rev..

[91] Punjamgod D., Kurinjery A., Annamalai M., Rathinam R., Kulanthaiyesu A. (2026). Structural diversity, biosynthesis, and extraction of brown algae fucoidan and its bio-stimulant applications in crop improvement. Crit. Rev. Biotechnol..

[92] Lee J.H., Yun C.W., Hur J., Lee S.H. (2018). Fucoidan Rescues p-Cresol-Induced Cellular Senescence in Mesenchymal Stem Cells via FAK-Akt-TWIST Axis. Mar. Drugs.

[93] Lee J.H., Lee S.H., Choi S.H., Asahara T., Kwon S.M. (2015). The Sulfated Polysaccharide Fucoidan Rescues Senescence of Endothelial Colony-Forming Cells for Ischemic Repair. Stem Cells.

[94] Mercurio D., Wagemaker T., Alves V., Benevenuto C., Gaspar L., Campos P.M. (2015). In vivo photoprotective effects of cosmetic formulations containing UV filters, vitamins, *Ginkgo biloba* and red algae extracts. J. Photochem. Photobiol. B Biol..

[95] Alkhalaf M.I. (2021). Chemical composition, antioxidant, anti-inflammatory and cytotoxic effects of *Chondrus crispus* species of red algae collected from the Red Sea along the shores of Jeddah city. J. King Saud. Univ.-Sci..

[96] Pohl F., Kong Thoo Lin P. (2018). The potential use of plant natural products and plant extracts with antioxidant properties for the prevention/treatment of neurodegenerative diseases: In vitro, in vivo and clinical trials. Molecules.

[97] Sridhar A., Ponnuchamy M., Kumar P.S., Kapoor A., Vo D.-V.N., Prabhakar S. (2021). Techniques and modeling of polyphenol extraction from food: A review. Environ. Chem. Lett..

[98] Hndeya A.G., Sbhatu D.B., Gebreyohannes G. (2026). Advances in Eco-Friendly Extraction of Fruit Bioactive Compounds: Technologies, Challenges and Future Directions. Anal. Sci. Adv..

[99] Wong F., Omori S., Donghia N.M., Zheng E.J., Collins J.J. (2023). Discovering small-molecule senolytics with deep neural networks. Nat. Aging.

[100] Pan Y., Cai H., Ye F., Xu W., Huang Z., Zhu J., Gong Y., Li Y., Ezemaduka A.N., Gao S. (2025). ElixirSeeker: A Machine Learning Framework Utilizing Fusion Molecular Fingerprints for the Discovery of Lifespan-Extending Compounds. Aging Cell.

[101] Santiago-de-la-Cruz J.A., Rivero-Segura N.A., Gomez-Verjan J.C. (2025). Structure-based machine learning screening identifies natural product candidates as potential geroprotectors. J. Cheminform..

[102] Zhang Z., Chen Y., Wang S., Chen G., Wang M., Pan Y., Li E. (2025). Prediction and analysis of anti-aging peptides using data augmentation and machine learning algorithms. BMC Biol..

[103] Kusumoto D., Seki T., Sawada H., Kunitomi A., Katsuki T., Kimura M., Ito S., Komuro J., Hashimoto H., Fukuda K. (2021). Anti-senescent drug screening by deep learning-based morphology senescence scoring. Nat. Commun..

[104] Duran I., Pombo J., Sun B., Gallage S., Kudo H., McHugh D., Bousset L., Avila J.E.B., Forlano R., Manousou P. (2024). Detection of senescence using machine learning algorithms based on nuclear features. Nat. Commun..

[105] He L., Li M., Wang X., Wu X., Yue G., Wang T., Zhou Y., Lei B., Zhou G. (2024). Morphology-based deep learning enables accurate detection of senescence in mesenchymal stem cell cultures. BMC Biol..

[106] Wall A., Kennedy K., Cal R., Casey R., Holton T., Adelfio A., Khaldi N. (2020). pep_35E7UW, a natural peptide with cutaneous anti-ageing effects discovered within the *Oryza sativa* proteome through machine learning. J. Dermatol. Cosmetol..

[107] Tennant N., Pavuluri A., Singh G., Cortez K., O’Connor-Giles K., Larschan E., Singh R. (2026). An snRNA-seq aging clock for the fruit fly head sheds light on sex-biased aging. Sci. Rep..

